# Transcriptome analysis revealed potential mechanisms of differences in physiological stress responses between caged male and female magpies

**DOI:** 10.1186/s12864-019-5804-0

**Published:** 2019-06-03

**Authors:** Yu Wang, Jinxin Guo, Lin Wang, Hengjiu Tian, Jinling Sui

**Affiliations:** 1 School of Nature Conservation, Beijing Forestry University, Qinghuadonglu No. 35, Haidian District, Beijing, 100083 China; 2 Beijing Wildlife Rescue Center, Shuanghelu No.1, Shunyi District, Beijing, 100029 China

**Keywords:** Blood transcriptome analysis, Differential gene expression, Environmental stress, Defense mechanism, Lifespan

## Abstract

**Background:**

Under caged conditions, birds are affected more severely by environmental stressors such as dietary structure, activity space, human disturbances, and pathogens, which may be reflected in the gene expression in peripheral blood or other tissues. Elucidating the molecular mechanism of these stress responses will help improve animal welfare.

**Results:**

In the present study, the blood transcriptomes of six male and five female caged magpies (*Pica pica*) were sequenced, and a total of ~ 100 Gb in clean reads were generated using the Illumina HiSeq 2000 sequencer. A total of 420,291 unigenes were identified after assembly, of which 179,316 were annotated in five databases, 7471 were assigned to 269 Kyoto Encyclopedia of Genes and Genomes (KEGG) pathways, and 566 were assigned to the Clusters of Orthologous Groups (COG) functional classification “defense mechanisms”. Analysis of differentially expressed genes (DEGs) showed that 2657 unigenes were differentially expressed between males and females (*q* < 0.1), and these DEGs were assigned to 45 KEGG pathways involving stress resistance, immunity, energy metabolism, reproduction, lifespan regulation, and diseases. Further analysis revealed that females might be more sensitive to stress through upregulation of c-Jun N-terminal kinases (JNKs) and 5’AMP-activated protein kinase (AMPK), and were also possibly more sensitive to dynamic changes in energy. Females expressed higher major histocompatibility complex (MHC) class II levels than males, enhancing resistance to pathogens, and the DEGs related to reproduction included MAPK, CaMK, CPEB, and Cdc25. The genes related to stress, energy, and immunity were also likely related to the regulation of longevity. The upregulated JNKs in females might prolong lifespan and relieve antioxidant stress. Females may also activate the AMPK pathway and implement dietary restrictions to prolong lifespan, whereas males may upregulate SIRT1 and CRAB to increase lifespan.

**Conclusions:**

Female magpies might be more sensitive to stress and dynamic changes in energy thus enhanced resistance to pathogens, and the genes related to stress, energy, and immunity were also possibly related to the regulation of longevity. Further confirmations with techniques such as RT-qPCR and western blot are necessary to validate the above arguments.

**Electronic supplementary material:**

The online version of this article (10.1186/s12864-019-5804-0) contains supplementary material, which is available to authorized users.

## Background

Mankind is currently transforming the natural environment at an unprecedented rate, and studies have demonstrated that wild animals are inevitably affected by the more and more urbanized environment [1, 2]. Environmental changes are stressors that induce individual organisms to respond physiologically as well as behaviorally [3–6]. Stress refers specifically to the way an animal’s body reacts to conditions such as threats, challenges, and physical and psychological disorders, and it can alter memory, reward and immune functions, metabolic patterns and intensity, and susceptibility to diseases [7]. Several studies have indicated that stress can cause diseases and decrease the adaptability of an organism; these studies have proposed to describe adaptability as a special resource, namely, adaptation energy [8, 9].

Numerous studies have reported that environmental changes led to alterations in the expression profiles of key genes associated with certain metabolic pathways in animals, such as the response of corals to seawater acidification [10], the tolerance of killifish to pollution [11], and the immune response of birds to malaria infection [12, 13]. Such alterations can be monitored and identified through transcriptome sequencing techniques, which have developed rapidly in recent years. Transcriptome analysis can reflect the real-time expression of related genes; by detecting the transcriptomic expression level of genes in a specific tissue, the genes and pathways related to specific phenotypic variations can be detected, and the response of phenotypic traits to environmental changes can be recorded.

The magpie (*Pica pica*), a passerine bird in the family Corvidae, is a resident breeding bird in both artificial (urban and rural) and natural habitats throughout northern parts of the Eurasian continent [14]. It is an ideal and rational experimental material or model for studying the adaptation of animals to artificial environments. Additionally, the large body size of magpies allows easier sampling of blood, feathers, and other tissues, compared to smaller birds [2]. Under caged conditions, magpies can be affected by stresses such as dietary structure, activity space, human disturbances, pathogens, noise pollution, and artificial night light. Previous studies have only considered differences in behavior and physiology caused by environmental changes among birds from distinct areas [2, 5, 6, 15, 16], and little attention has been given to differences between males and females of the same species.

There are several advantages in studying blood transcriptomes, as peripheral blood has multiple functions. First, peripheral blood is relatively easier to acquire than other tissues and often causes less damage to the sampled animal, which is particularly relevant for the study of wild animals. Second, blood performs many important functions within the body and is useful in studying a wide spectrum of physiological functions such as stress response, energy metabolism, and immunity. Therefore, a comprehensive understanding of transcriptome levels in bird blood is indispensable for understanding birds’ stress responses to diseases [12, 17].

In the present study, we focused on the differences in behavior and physiology between male and female magpies under caged conditions by performing a de novo assembly of all clean reads from blood from 11 magpies, using the Trinity platform. Many unigenes were matched to avian genes from birds such as the crow (*Corvus* sp.) and great tit (*Parus major*) by database mapping, indicating that the assembly of unigenes by Trinity was accurate and reliable. The transcription levels of genes in the whole blood of the caged male and female magpies were identified and analyzed by high-throughput RNA sequencing technology (Illumina RNA-seq) to determine the expression levels of genes related to stress resistance, immunity, energy metabolism, reproduction, and disease. Using whole-blood transcriptome data, we first performed functional annotations on the blood genes of the caged magpies, and then determined and compared the differences in gene expression between the male and female magpies.

## Results

### Sequencing and de novo assembly of unigenes

A total of 716 million raw paired-end reads were obtained using the Illumina HiSeq 2000 sequencer from 11 samples of magpie blood, reaching a total length of 101 Gbp. After removing adaptor sequences, filtering out reads containing > 1% ambiguous bases (Ns), and trimming low-quality sequences (quality score, Q < 30), 705 million clean paired-end reads with a total length of 100 Gbp remained, which were subsequently mixed and pooled into one de novo transcriptome database. The average GC content and sequence length of the de novo transcriptome database were 51% and 147 bp, respectively. As shown in Table 1, assembly of the clean reads using the Trinity platform under the default settings resulted in 420,291 unigenes, ranging from 201 to 22,706 bp, with an average size of 503 bp.

**Table 1:** Length distribution of all unigenes

Length (bp)	Total	≤200	201–500	501–1000	1001–1500	1501–2000	> 2000
Number	420,291	0	255,537	90,062	29,045	14,561	31,085
%	100	0	60.80	21.43	6.91	3.46	7.40

**Fig. 1:**
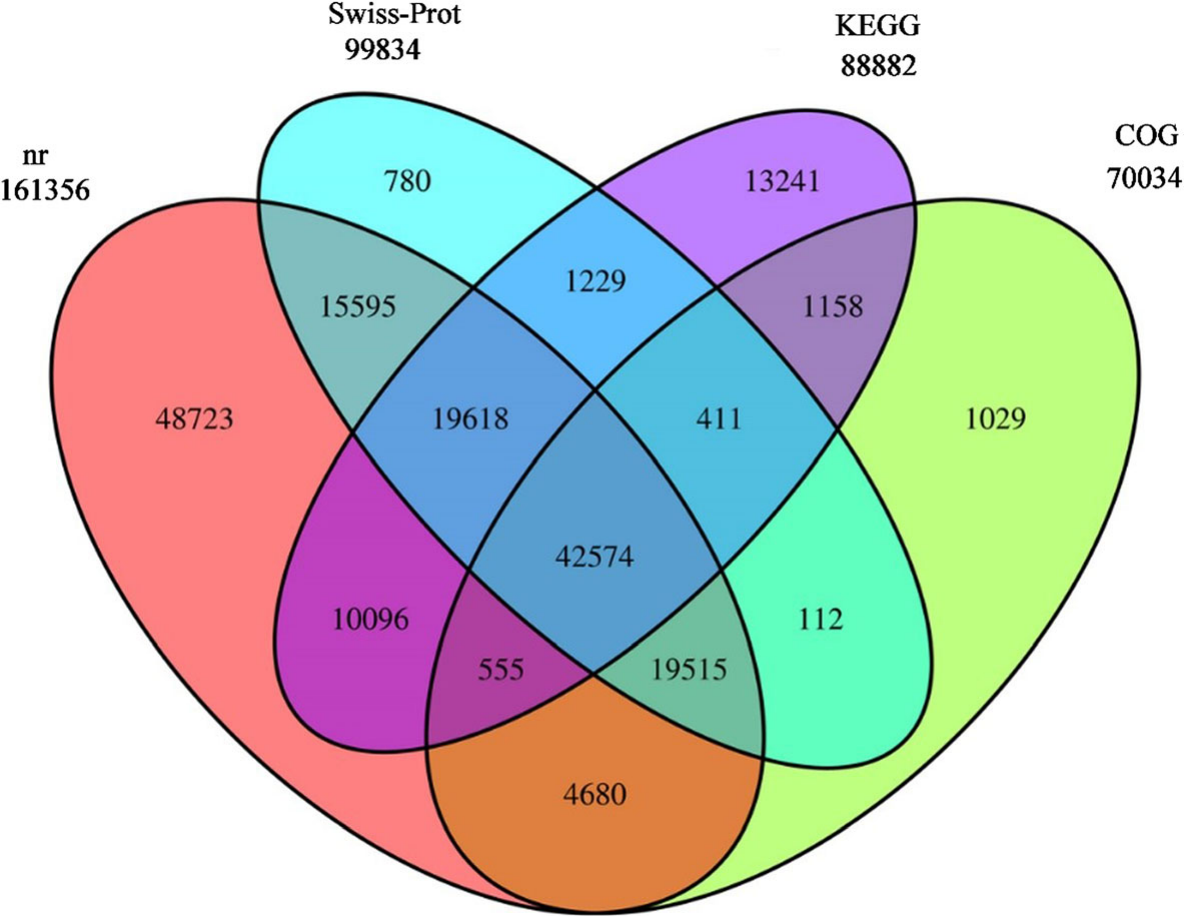
Venn diagram showing the number of unigenes annotated in the nr, COG, Swiss-Prot, and KEGG protein databases. Overlapping sections indicate unigenes that were annotated in two or more databases; non-overlapping sections indicate unigenes annotated in only one database

Figure 1 shows the breakdown of the 179,316 total unigenes that were annotated in the NCBI nr (National Center for Biotechnology Information nonredundant; 161,356 unigenes), Swiss-Prot (Swiss-Prot/UniProtKB, the universal protein resource, a central repository of protein data created by combining the Swiss-Prot, TrEMBL and PIR-PSD databases; 99,834), KEGG (Kyoto Encyclopedia of Genes and Genomes; 88,882), and COG (Clusters of Orthologous Groups; 70,034) databases, of which 42,574 unigenes were annotated in all four databases, accounting for 23.74% of the total annotated unigenes. In addition, 20,458 unigenes were annotated in the GO (gene ontogeny) database.

In total, 18,230 potential simple sequence repeats (SSRs; also known as microsatellites) were identified in 17,879 unigenes (4.25% of the total 420,291 unigenes); 696 of these unigenes contained more than one SSR (Table 2). Among the SSRs, the most abundant motifs were mononucleotide repeats (90.0%); penta-, hexa-, and octanucleotide repeats appeared with very low frequency (totaling 2‰). The most common motifs were A/T mononucleotide SSRs, accounting for 90% of the total. There were many variations in the transcriptome among magpie individuals, and the degree of variation of these genes may lead to phenotypic differences among individuals, such as resistance to disease, stress, etc. These phenotypic differences might then affect the fitness of individuals against various adverse environments.

**Table 2:** SSRs in unigenes

SSRs	monoN^a^	diN	triN	quadN	pentaN	hexaN	octaN
Number	16,407	765	905	115	19	18	1

^a^*N* nucleotide

### Unigenes annotated in the nr and Swiss-Prot protein databases

**Fig. 2:**
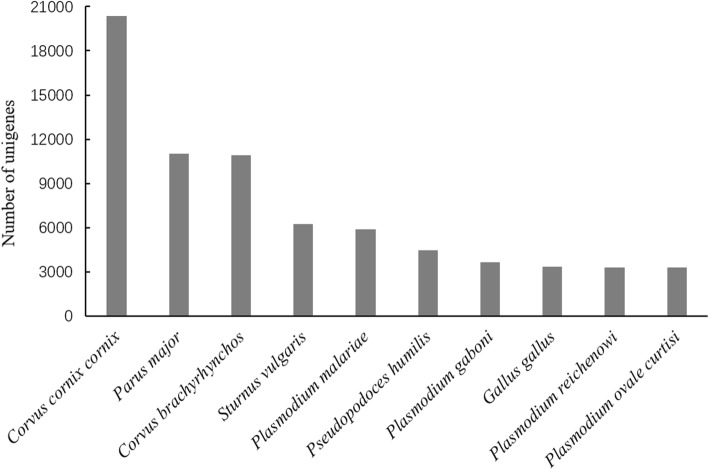
Top 10 species distribution from the nr functional annotation

First, the identified unigenes were queried against the nr and Swiss-Prot protein databases to acquire species information. As shown in Fig. 1, there were 161,356 unigenes annotated in the nr database, of which 31,311 unigenes were matched to *Corvus cornix cornix* and other bird species with a minimum similarity threshold of *e* < 10^− 5^ (Fig. 2). Figure 2 also presents the top 10 species matched in the nr database, six of which are bird species and four of which belong to the genus *Plasmodium*; in total, 72,446 unigenes were matched to these ten species. The most represented species was *C. cornix cornix* (20,384 unigenes), followed by *Parus major* (11,012 unigenes), *Corvus brachyrhynchos* (10,899 unigenes), and *Sturnus vulgaris* (6223 unigenes). Only 3349 unigenes were annotated to *Gallus gallus*, and only 4474 unigenes were annotated to *Pseudopodoces humilis*. The four *Plasmodium* species in the top 10 were represented by a total of 16,105 unigenes. There were 99,834 unigenes annotated in the Swiss-Prot protein database; most were matched to mammals such as human (33%), mouse (20%), and cow (8%), but the domestic chicken (*Gallus Gallus domesticus*) was also represented (10%).

### Unigenes annotated in the COG, GO, and KEGG functional databases

The identified unigenes were subsequently queried against the COG, GO, and KEGG databases for functional category analysis (Additional file 1: Figure S1). There were 70,034 unigenes aligned with 26 COG functional classifications; the largest group was “signal transduction mechanisms” (10,263 unigenes), followed by “general function prediction only” (9257), “post translational modification, protein turnover, chaperone function” (7681), and “transcription” (5962) (Additional file 1: Figure S1A). Notably, 566 unigenes were assigned to the classification “defense mechanisms (V)”, which may be associated with the immunity or adaptation energy of birds to various environmental stressors.

GO analysis annotated 87,107 unigenes that were assigned to three main categories: “biological process” (32,901 unigenes), “molecular function” (25,854), and “cellular component” (28,352) (Additional file 1: Figure S1B). The six major terms in the “cellular component” category were “cell part” (8560), “organelle” (5520), “macromolecular complex” (4496), “organelle part” (4052), “membrane part” (2592), and “membrane” (1679). Under the “molecular function” category, “binding” (12,053), “catalytic activity” (9253), “structural molecule activity” (1183), and “transporter activity” (1135) were the four dominant terms, the largest two being typical of transcriptomic studies regardless of the taxa or tissue type sequenced [18–20]. Under the “biological process” category, most unigenes were involved in the seven following terms: “cellular process” (7916), “metabolic process” (7547), “single-organism process” (5258), “biological regulation” (4942), “localization” (2030), “developmental process” (1645), and “response to stimulus” (1436); this is similar to findings from kiwi bird blood [21]. Although the number of unigenes assigned to the “immune system process” term in the “biological process” category was not large (395 unigenes), they were largely implicated in bird immunity.

KEGG analysis was performed to address the unigenes associated with biological pathways (Additional file 1: Figure S1C). A total of 7471 of the 88,882 unigenes were assigned to 269 KEGG pathways belonging to six functional categories: “human disease” (71,646), “organismal systems” (54,699), “environmental information processing” (39,175), “metabolism” (33,450), “genetic information processing” (27,701) and “cellular processes” (27,267). Most unigenes in the “environmental information processing” category were assigned to pathways under the “signal transduction” term (35,332, accounting for 90.19% of the total in this category), although this was not the most represented main category. The “infectious diseases: viral” term (15,556) was the largest group in the “human diseases” category, and “endocrine system” (14,875) and “immune system” (14,867) where the largest groups in the “organismal systems” category. The functions of these unigenes may reflect the relationship between the magpie and its environment, and functions related to the immune system and disease infection may further indicate the magpie’s resistance to disease stress and the dynamic relationship between host and pathogen.

### Identification and analyses of differentially expressed genes (DEGs)

**Fig. 3:**
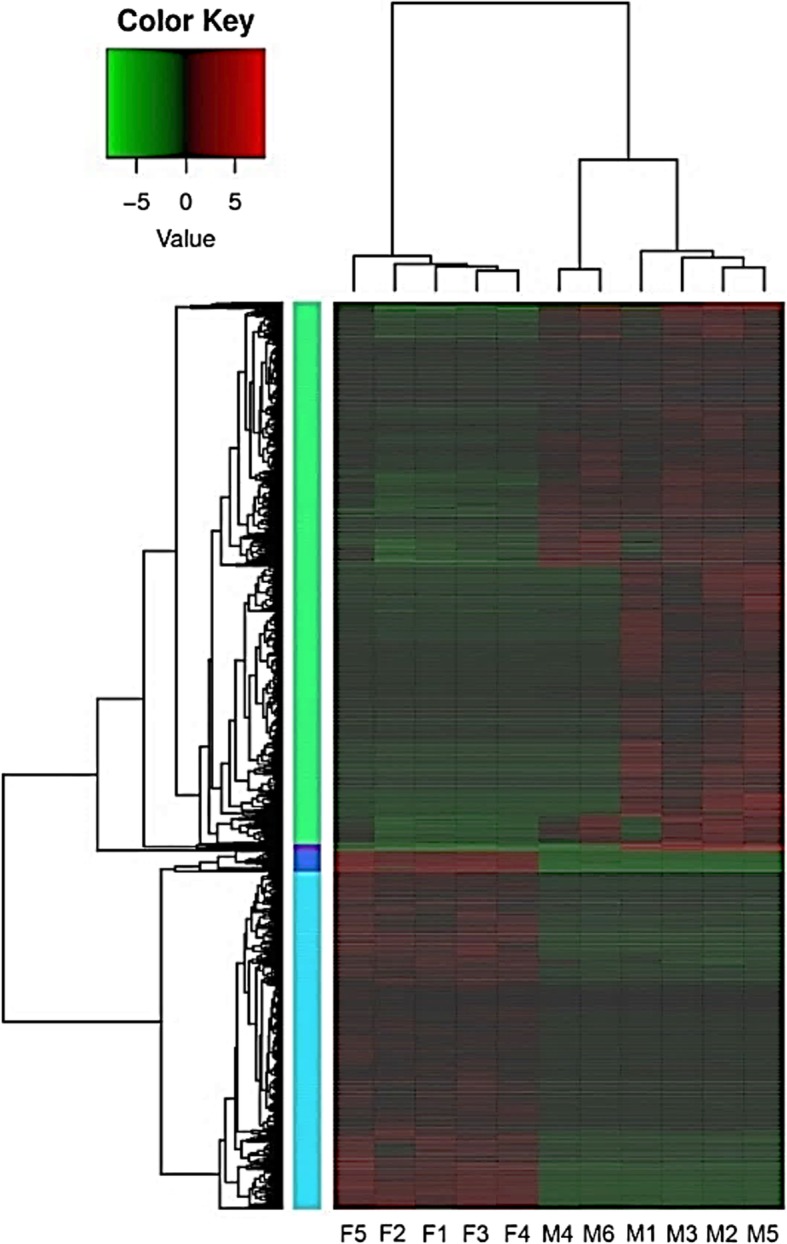
Heatmap showing expression levels (log_2_FPKM) of the 2658 differentially expressed genes (DEGs) between male and female magpies. Individual magpies are denoted F1–F5 and M1–M6 for females and males, respectively. Log_2_FPKM values of individual DEGs were used for cluster analysis. Red indicates higher expression and green indicates lower expression, as indicated by the scale in the upper left corner

**Fig. 4:**
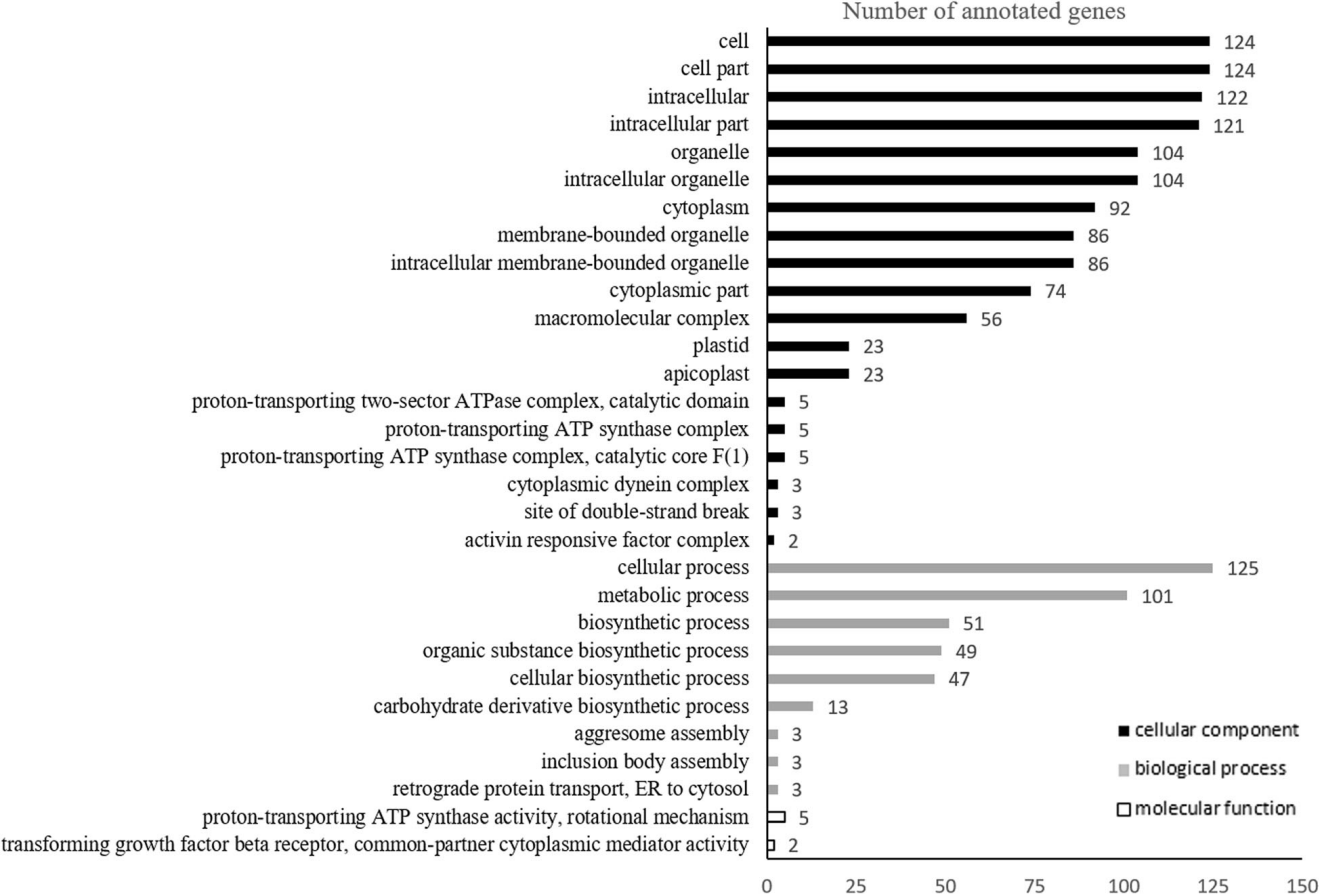
Distribution of the DEGs annotated with Gene Ontology (GO) terms

Identification of DEGs revealed that male and female caged magpies exhibited significantly different gene expression profiles. A heatmap illustrating the expression levels of different genes (rows) for male and female magpies was generated using data from the blood samples of 11 individual magpies (5 females and 6 males) (Fig. 3). By mapping reads to the reference and counting the number of reads mapped to each transcript, we found that 2657 unigenes in blood were significantly differentially expressed between females and males (log_2_FC range from − 12 to 8, *q* < 0.1; Fig. 4), while the remaining unigenes, which may be housekeeping genes, showed no significant differential expression.

The heatmap shows that unigenes with similar expression patterns were mainly clustered into two major branches on the y-axis (Fig. 3). The upper major branch included 1608 unigenes, and could be further divided into two sub-branches. One sub-branch contained 1588 unigenes with log_2_FPKM values (fragment per kilobase of exon per million reads mapped) from 1 to 8.9, of which 367 unigenes were expressed 2-fold more (i.e., 1 < log_2_FPKM < 2) in males than in females, with M4 and M6 as exceptions (more than 800 unigenes downregulated due to individual differences). These unigenes might be located on the male Z chromosome (note: the male has two ZZ chromosomes). Of the 1588 unigenes, 1013 were annotated to the nr database, 564 to Swiss-Prot, 487 to COGs, 137 to GO terms, and 39 to KEGG pathways. The other sub-branch contained 20 unigenes with log_2_FPKM values from 3 to 7.8; among them, 19 unigenes were upregulated in male magpies and one was upregulated in females. Two of the 20 unigenes were annotated to the nr database, one to Swiss-Prot, one to a GO term, and one to a KEGG pathway.

As shown in Fig. 3, the lower major branch of the tree included 1049 unigenes with log_2_FPKM values from 1 to 12, and was further divided into two sub-branches. One sub-branch contained 81 unigenes with log_2_FPKM values from 3 to 12, with 20 downregulated and 61 upregulated in females, 20 upregulated and 61 downregulated in M1, M2, M3, and M5, and all 81 downregulated in M4 and M6. Of these 81 unigenes, 43 were annotated to the nr database, 23 to Swiss-Prot, 16 to COGs, 8 to GO terms, and 14 to KEGG pathways. The other sub-branch contained 988 unigenes with log_2_FPKM values from 1 to 10.7; these unigenes were upregulated in female magpies and downregulated in males. Of the 988 unigenes, 682 were annotated to the nr database, 402 to Swiss-Prot, 189 to COGs, 40 to GO terms, and 158 to KEGG pathways. The heatmap analysis showed that in general, more unigenes were upregulated in the blood of male magpies than in that of female magpie.

As shown in Fig. 4, the GO enrichment analysis revealed that of the 2657 genes that were differentially expressed between males and females in blood transcriptomes (*q* < 0.1), 1564 could be assigned to 30 significantly enriched GO terms in the categories “cellular components” (1162 DEGs), “biological processes” (395), and “molecular functions” (7). Genes related to the “cellular components” category were primarily categorized into cell (124 genes; GO: 0005623), cell part (124 genes; GO: 0044464), intracellular (122 genes; GO: 0005622), intracellular part (121 genes; GO: 0044424), intracellular organelle (104 genes; GO: 0043229), and organelle (104 genes; GO: 0043226); those in the “biological process” category were categorized into cellular processes (125 genes; GO: 0009987) and metabolism processes (101 genes; GO: 0008152); and those in the “molecular functions” category were categorized into proton-transporting ATP synthase activity and rotational mechanism (5 genes; GO: 0046933) and transforming growth factor beta receptor and common-partner cytoplasmic mediator activity (2 genes; GO: 0030616). The distribution of GO annotations in different functional categories indicated substantial diversity of DEGs (Fig. 4).

We also performed KEGG pathway analysis of these DEGs and found that they were annotated to 45 of the 269 KEGG pathways (*q* < 0.1). Of these 46 KEGG pathways, several were involved in functions of interest for this study, including immunization (2 pathways), energy metabolism (8 pathways), reproduction (3 pathways), and disease (12 pathways), as these genes may be associated with the fitness of birds. In the following two sections, we discuss the functional significance of these DEGs in female and male magpies.

## Discussion

### Female magpies may be more susceptible to stress than male birds

**Fig. 5:**
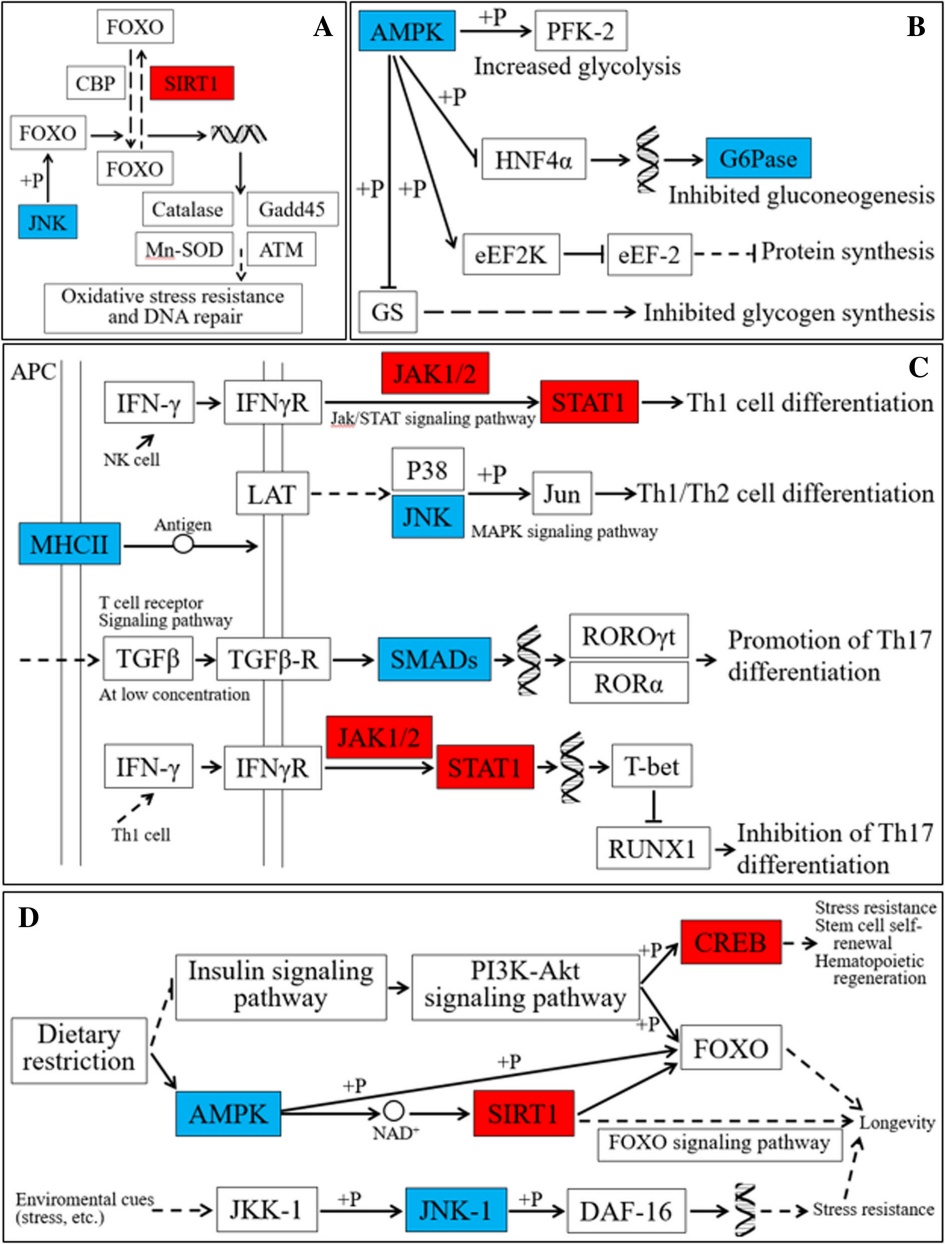
Functional significance of several key genes from four KEGG pathways differentially expressed in female and male magpies. The proteins encoded by genes marked in blue were upregulated in female birds, whereas genes upregulated in male birds are in red. **a** The relationship between the key DEGs and stress resistance excerpted from the FOXO signaling pathway (ko04068) shown in Additional file 2: Figure S2; the JNK stress resistance gene was upregulated in females, and the SIRT1 stress resistance gene was upregulated in males. **b** The relationship between the key DEGs and energy metabolism excerpted from the AMPK signaling pathway (ko04152) shown in Additional file 3: Figure S3; females enhanced AMPK and G6Pase to regulate energy metabolism. **c** The relationship between the key DEGs and the immune system excerpted from the Th1 and Th2 cell differentiation pathway (ko04658) and the Th17 cell differentiation pathway (ko04659) shown in Additional file 4: Figure S4A and S4B, respectively; females upregulated MHC II, JNK, and SMADs to promote the differentiation of Th1/Th2 and Th17 cells, whereas males inhibited the differentiation of Th17 cells through Th1 cells. **d** The relationship between key DEGs and longevity excerpted from the longevity regulating pathway (ko04211) and the longevity regulating pathway worm (ko04212) shown in Additional file 6: Figure S5A and S5B, respectively; females regulated longevity through the AMPK energy metabolism and JNK stress resistance pathways, whereas males regulated longevity through CREB and SIRT1 stress resistance genes. Boxes represent signal pathways or gene products (i.e., proteins or RNA); solid arrows represent interactions and relationships between molecules; dotted arrows represent indirect or unknown relationships; the double helix represents DNA, circles represent molecules as indicated, and + P represents the process of phosphorylation. JNK, c-Jun *N*-terminal kinase; FOXO, forkhead box protein; CBP, E1A/CREB-binding protein; Cadd45, growth arrest and DNA-damage-inducible protein; Mn-SOD, Mn-superoxide dismutase; ATM, serine-protein kinase ATM; AMPK, 5′-AMP-activated protein kinase; PFK-2, 6-phosphofructo-2-kinase; HNF4α, hepatocyte nuclear factor 4α; G6Pase, glucose-6-phosphatase; eEF2K, elongation factor 2 kinase; eEF-2, elongation factor 2; GS, glycogen synthase; MHCII, major histocompatibility complex, class II; APC, antigen-presenting cells; NK cells, natural killer cells; IFNγ, interferon-γ; IFNγR, interferon-γ receptor; JAK1/2, Janus kinase 1/2; STAT1, signal transducer and activator of transcription 1; LAT, linker for activation of T-cells; P38, p38 MAP kinase; Jun, transcription factor AP-1; TGF-β, transforming growth factor β: TGFβ-R, TGF-β receptor; SMADs, mothers against decapentaplegic homolog; RORγt, RAR-related orphan receptor γ; RORα, RAR-related orphan receptor α; T-bet, T-box protein; RUNX1, runt-related transcription factor 1; CREB, cyclic AMP-responsive element-binding protein; JKK-1, dual specificity mitogen-activated protein kinase kinase JKK-1; and DAF-16, forkhead box protein O3

We found that genes encoding c-JunN-terminal kinases (JNKs), stress sensors responsive to stress stimuli, were upregulated in female magpies (Fig. 5 A). JNK is activated by stress, thus its upregulation indicates that female birds might be more susceptible or responsive to stress and thus might experience more stress than their male counterparts. Meanwhile, male birds not only downregulated JNK, but also significantly upregulated genes encoding silent mating type information regulation 2 homolog 1 (sirtuin 1, SIRT1; Fig. 5 A), an enzyme that plays a key role in stress resistance and longevity [22]. This result indicates that male birds may possess higher stress resistance than female birds. In addition, these two sets of genes (i.e., genes encoding JNK and SIRT1) were significantly enriched in the FOXO signaling pathway (ko04068, *q* < 0.01, Fig. 5 A), which is related to oxidative stress resistance, longevity, apoptosis, cell-cycle control, and glucose metabolism [23–25]. Therefore, it is speculated that JNK protein kinase may activate the FOXO transcription factor through phosphorylation, and the SIRT1 enzyme may modify FOXO via deacetylation after translation. Activated FOXO protein would enter the nucleus and bond to DNA to regulate expression of genes associated with stress resistance, apoptosis, glucose metabolism, and lifespan (Fig. 5a and Additional file 2: Figure S2).

### Female magpies might be more sensitive to energy dynamics

As shown in Fig. 5b, compared to their male counterparts, female magpies had significantly upregulated expression of AMPK (Log_2_FC = 3, *q* < 0.01) in the AMPK signaling pathway (ko04152). AMPK (i.e., 5’AMP-activated protein kinase) is a sensor of cellular energy status and plays a key role in energy homeostasis, and largely activates the uptake and oxidation of glucose and fatty acids, inhibits syntheses of gluconeogenesis, glycogen, and protein, and stimulates glycolysis when cellular energy is low (Fig. 5b) [26]. Upregulation of AMPK in female birds suggests that they were possibly susceptible to energy dynamics in vivo, thereby requiring rapid energy input when encountering energy consumption events such as starvation, hypoxia, intense muscle contraction, and tension. Indeed, female birds also upregulated G6PCase (glucose-6-phosphate carboxylase; Fig. 5b), an enzyme functioning in and glycogenolysis [27], which plays roles not only in the AMPK signaling pathway but also in the insulin resistance, glucagon, insulin, adipocytokine, and PI3K-Akt signaling pathways. Downregulation of these two genes in male magpies probably suggests that the male birds hold higher energy homeostasis than their female counterparts, and therefore may be more resistant to vigorous energy disorders. Thus, under the same stress conditions, female birds may be required to generate more energy via gluconeogenesis and glycogenolysis with upregulated AMPK and G6Pase to adapt to energy dynamics induced by stress resistance (Fig. 5b and Additional file 3: Figure S3).

### Female magpies expressed higher levels of MHC class II than male birds

Figure 5c shows that, compared to their male counterparts, female magpies exhibited significantly upregulated major histocompatibility complex (MHC) class II (Log_2_FC = 3) in the Th17 cell differentiation (ko04659) and Th1 and Th2 cell differentiation pathways (ko04658). MHC class II is an important class of molecules found only in antigen-presenting cells (APCs) and can recognize, bind, and present foreign antigens to T-cells [28], making it critical for immunity. These results imply that female magpies might resist bacteria both inside and outside the cell by upregulating the expression of MHC class II molecules and promoting differentiation of Th1 and Th17 cells, whereas excessive Th1 and Th17 cells could possibly cause autoimmune diseases. In contrast, the cytokine interferon-γ produced by Th1 cells could possibly inhibit the differentiation of Th17 cells through JAK1/2 (Janus kinase1/2; interacts with multiple major cytokine receptors) and STAT1 (signal transducer and activator of transcription 1; a transcription factor involved in upregulating gene transcription) cascades, thus male magpies may activate Th1 cells by upregulating JAK1/2 and STAT1 cascades to inhibit excessive Th17 cell differentiation and production of autoallergic responses (Fig. 5c and Additional file 4: Figure S4B and S4A).

Indeed, numerous DEGs were significantly enriched in pathways related to various diseases (Table 3). These diseases are almost entirely caused by pathogens such as viruses and bacteria. In accordance with these hypotheses, MHC class II in female magpies (Fig. 5c) was also upregulated in herpes simplex infection (ko05168), tuberculosis (ko05152), leishmaniasis (ko05140), and systemic lupus erythematosus (ko05322) pathways. Thus, under the same pathogen stress, we speculate that the female magpies may possess lower tolerance than males, and therefore express higher levels of MHC class II to increase immunity (Fig. 5C and Additional file 5: Figure S6A–S6D).

**Table 3:** Expression of disease-related DEGs in female vs. male magpies

Disease	DEG	Regulation in female magpies
Herpes simplex infection	MAPK	UP
	MCH2	UP
	CD74	UP
	HNRNPK	UP
	PPP1C	DOWN
	EIF2S1	DOWN
	JAK	DOWN
	STAT	DOWN
	MHC1	DOWN
Tuberculosis	MCH2	UP
	CAMK2	UP
	CD74	UP
	CTSS	UP
	MAPK	UP
	JAK	DOWN
	STAT	DOWN
	PIK3C3	DOWN
	RAB5A	DOWN
	LAMP	DOWN
	PPP3C	DOWN
Leishmaniasis	MCH2	UP
	JAK	DOWN
	STAT	DOWN
Legionellosis	NFKB2	UP
Systemic lupus erythematosus	MHC2	UP
	C5	UP
	C7	UP
	H2A	DOWN
	H3	DOWN
	H4	DOWN
Non-alcoholic fatty liver disease	PRKAA	UP
	MAPK	UP
	EIF2S1	DOWN
	NDUFV1	DOWN
Alzheimer’s disease	ATP5F1A	UP
	NDUFV1	DOWN
	PPP3C	DOWN
	CDK5	DOWN
	CAPN1	DOWN

### Genes related to reproduction and longevity are regulated differently in female and male magpies

As shown in Table 4, several candidate genes related to reproduction were differentially expressed in female and male magpies. For example, female birds upregulated MAPK (mitogen-activated protein kinase) and CaMK (Ca^2+^/calmodulin-dependent protein kinase), whereas male birds upregulated genes encoding CPEB (cytoplasmic polyadenylation element binding protein), Cdc25 (M-phase inducer phosphatase 2), CALN (calcium-binding protein), and MLCP (myosin light-chain phosphatase) (Table 4).

**Table 4:** DEGs related to reproduction

Gene	Protein	Definition	Function	Male	Female
MAPK	MAPK	mitogen-activated protein kinase	Regulation of cell proliferation Gene expression Differentiation Mitosis Cell survival Apoptosis	down	up
CaMK	CaMK	calcium/calmodulin-dependent protein kinase	Regulation of gene expression	down	up
CPEB	CPEB	cytoplasmic polyadenylation element binding protein	Spermatogenesis Formation of long-term memory	up	down
Cdc25B	Cdc25	dual-specificity phosphatase	Control of cell cycle	up	down
CALN	CALN	calcium-binding protein	Binding of Ca^2+^	up	down
PPP1C	MLCP	myosin phosphatase	Muscle contraction	up	down

Interestingly, several DEGs were significantly enriched in pathways associated with lifespan regulation (Fig. 5d). Regulation of longevity depends on genetic as well as environmental factors. The JNK protein, responsive to stress stimuli and upregulated in female magpies (Fig. 5a and d), was found to play a role in extending lifespan in the roundworm *Caenorhabditis elegans* via oxidative stress resistance and resistance to other stresses [29, 30]. Recent studies have also shown that dietary restriction, that is, limiting food (fatty acid, glucose, and protein) intake, substantially increased the healthy lifespan of laboratory model organisms [31]. Four pathways have been implicated in mediating this dietary restriction effect, including the insulin like growth factor (IGF-1)/insulin signaling pathway (ko04910), the sirtuin pathway (ko04211), the adenosine monophosphate (AMP) activated protein kinase (AMPK) pathway (ko04152), and the target of rapamycin (TOR) pathway (ko04150). In our current study, female magpies upregulated AMPK in the AMPK pathway (Fig. 5b and d), suggesting that they may mediate dietary restriction through the AMPK pathway (Additional file 6: Figure S5C, S5A, Additional file 3: Figure S3, Additional file 6: Figure S5D).

At the same time, male magpies upregulated the SIRT1 (sirtuin 1) gene (Figs. 5A and D); SIRT1 is another enzyme contributing to extended longevity (Fig. 5a) [22, 32]. This implies that male magpies may regulate dietary restriction via the sirtuin pathway. The collective response of these pathways to dietary restriction is believed to promote cellular fitness and ultimately longevity via activation of autophagy, stress defense mechanisms, and survival pathways, while attenuating pro-inflammatory mediators and cellular growth [33, 34]. Of course, the effect of dietary intake control on longevity can only be reflected by long-termfollow-up records, and speculation based solely on transcriptome data may not be sufficient.

The above results indicate that magpies may increase fitness and prolong lifespan by regulating dietary intake and through upregulation of several stress resistance genes when subjected to adverse effects such as oxidative stress, disease stress, human interference stress, and so on. In addition, male magpies also upregulated the gene encoding the CREB protein, a transcription factor that regulates the expression of genes associated with stress resistance, stem cell self-renewal, and hematopoietic regeneration after binding to DNA. These genes are also associated with regulating lifespan (Fig. 5d).

## Conclusions

To our knowledge, this is the first publically available transcriptome resource for the magpie. Our study is the first example of the use of Illumina paired-end sequencing technology to investigate the whole blood transcriptome of the magpie and compare differences in gene expression between the sexes. As dosage compensation has not yet been found in the avian sex determination system [35], we cannot determine whether up- or downregulated genes in male or female magpies contribute to equality of gene expression from different sex chromosomes or represent sex-biased expression, as a number of studies have shown sex-linked expression patterns in genes located on autosomes [36]. Thus, future efforts should determine whether the DEGs in the two sexes are caused by dosage compensation or sex-biased expression.

Transcriptome analysis can provide a critical complement to understanding an organism’s genome. This is one of the first studies to quantify variation in genome-wide gene expression of caged birds through blood transcriptome analysis of female and male magpies. The caged magpies were affected by dietary structure, activity space, human disturbances, etc., and thus exhibited differences in gene expression. The DEGs were involved in stress resistance, immunity, energy metabolism, disease, reproduction, and longevity. The results showed that the female birds upregulated the stress sensor gene JNK, implying that female magpies may possess low tolerance to stress and might be more easily affected by stress than their male counterparts. Furthermore, female magpies might also be sensitive to energy dynamics due to the upregulated AMPK. Compared to their male counterparts, female magpies exhibited higher expression levels of MHC class II, which is responsive to pathogens, indicative of their sensitive immune response. Female magpies are sensitive to stress, energy, and pathogens, suggesting that they require a more peaceful, cleaner breeding environment and a more reasonable diet than male magpies. Additionally, male and female magpies appear to regulate longevity possibly by mediating genes in the AMPK and sirtuin pathways, respectively. Obviously, further confirmations with techniques such as RT-qPCR and western blot are necessary to validate the above arguments.

## Methods

### Birds and blood sampling

All 11 magpies, five females and six males, were rescued from the wild by the Beijing Wildlife Rescue Center in 2016. The sex of each individual was identified using RT-PCR (primers sex1: 5′-CTCCCAAGGATGACTGTGCAAAACAG-3′; sex2: 5′-CCTTCACTTCCATTAAAGCTGATCTGGAATC-3′) [37]. All birds were fledglings; each individual was healthy, raised in a 90 × 60 × 50 cm^3^ cage, and fed fruits, forage, and yellow mealworms (*Tenebrio molitor*). All cages were kept in the same room so that the birds could see each other; they were fed by one person and lived with reasonable daytime light. The male and female birds were denoted as M1–M6 and F1–F5, respectively. The 11 blood samples, 15 μL each, were collected from the brachial vein into TRIzol tubes (Invitrogen, Carlsbad, CA, USA) on 5 May 2017, during the breeding season, and immediately stored in liquid nitrogen for use in subsequent RNA sequencing. After samples collection, all the magpies were released to the wild after they were confirmed by the veterinarian of Beijing Emergency Center to meet the health standards for release.

### RNA extraction, library construction, and sequencing

Total RNA was extracted from the 15-μL whole blood samples using TRIzol Reagent (Invitrogen) according to the manufacturer’s protocol. RNA degradation and contamination were assessed in 1% agarose gels, and RNA concentrations were determined quantitatively with the Agilent 2100 Bioanalyzer (Agilent Technologies, Palo Alto, CA, USA) and qualitatively with the NanoDrop spectrometer (Thermo Fisher Scientific, Waltham, MA, USA). A total of 1 μg RNA with an RNA integrity number (RIN) > 7 was used as input material for each library construction.

Next-generation sequencing libraries were constructed according to the manufacturer’s protocol (NEBNext Ultra RNA Library Prep Kit for Illumina, New England BioLabs, Ipswich, MA, USA). The poly(A) mRNA isolation was performed using the Ribo-Zero rRNA Removal Kit (Illumina, San Diego, CA, USA). The mRNA fragmentation and priming were performed using NEBNext First Strand Synthesis Reaction Buffer and NEBNext Random Primers. The first strand cDNA was synthesized using ProtoScript II Reverse Transcriptase, and the second strand cDNA was synthesized using the Second Strand Synthesis Enzyme Mix. Libraries with different indices were multiplexed and loaded on an Illumina HiSeq instrument according to the manufacturer’s instructions (Illumina). Sequencing was performed using a 2 × 150 bp paired-end (PE) configuration; image analysis and base calling were conducted using the HiSeq Control Software (HCS) + OLB + GAPipeline-1.6 (Illumina) on the HiSeq instrument.

### Transcriptome analysis

After sequencing, raw reads were cleaned by removing adapter and low-quality sequences using Cutadapt (version 1.9.1; Department of Computer Science, TU Dortmund, Germany) [38]. The obtained clean reads were subsequently assembled as unigenes using Trinity (version 2.2.0) [39], a novel method for the efficient and robust de novo reconstruction of transcriptomes from RNA-seq data. Duplicate contigs were removed using CD-HIT. With the unigenes sequences as a reference genes file, the expression levels of genes and their isoforms were estimated using RSEM (version 1.2.6; Madison, WI, USA) [40] from the clean paired-end data. We did not use the known zebra finch (*Taeniopygia guttata*) genome as the reference, owing to the low alignment between the zebra finch and magpie [21, 41, 42].

### Analyses of DEGs

DEGs were analyzed using the DESeq Bioconductor package (European Molecular Biology Laboratory; EMBL, Heidelberg, Germany) [43], a model based on the negative binomial distribution. *P*-values were corrected for false positives using the Benjamini and Hochberg false discovery rate (FDR) correction for multiple testing. Genes with an FDR (or *q*-value) < 0.1 were regarded as significantly differentially expressed, and thus as DEGs.

### GO and KEGG enrichment analyses

GO-Term Finder was used to identify Gene Ontology (GO) terms that annotate a list of enriched genes with a significant *P*-value < 0.05. The Kyoto Encyclopedia of Genes and Genomes (KEGG) is a collection of databases dealing with genomes, biological pathways, diseases, drugs, and chemical substances (https://www.kegg.jp/). We used in-house scripts to find KEGG pathways enriched in DEGs.

All unigene sequences were also annotated using BLAST against the nr, Clusters of Orthologous Genes (COG), Swiss-Prot, KEGG, and GO databases.

## Additional files

Additional file 1: **Figure S1.** Unigenes function categories. (A) COG function categories; (B) All unigenes KEGG barplot; (C) All unigenes Go Bar. (PNG 229 kb)

Additional file 2: FOXO signaling pathway (ko04068). Genes colored in red and blue represent that they were up-regulated in male and female magpies, respectively, and those colored in green indicate that expressions of the genes were either up- or down-regulated in both male and female magpies. (PNG 23 kb)

Additional file 3: The AMPK signaling pathway (ko04152). Genes colored in red and blue represent that they were up-regulated in male and female magpies, respectively, and those colored in green indicate that expressions of the genes were either up- or down-regulated in both male and female magpies. (PNG 33 kb)

Additional file 4: **Figure S4A**. The Th1 and Th2 cell differentiation pathway (ko04658 ). **Figure S4B.** The Th17 cell differentiation pathway (ko04659 ). Genes colored in red and blue represent that they were up-regulated in male and female magpies, respectively, and those colored in green indicate that expressions of the genes were either up- or down-regulated in both male and female magpies. (ZIP 62 kb)

Additional file 5: **Figure S5A.** The longevity regulating pathway (ko04211). **Figure S5B.** The longevity regulating pathway-worm (ko04212 ). **Figure S5C.** The insulin signaling pathway (ko04910). **Figure S5D.** The mTOR signaling pathway (ko04150). Genes colored in red and blue represent that they were up-regulated in male and female magpies, respectively, and those colored in green indicate that expressions of the genes were either up- or down-regulated in both male and female magpies. (ZIP 93 kb)

Additional file 6: **Figure S6A.** The herpes simplex infection pathway (ko05168). **Figure S6B.** The tuberculosis pathway (ko05152). **Figure S6C.** The leishmaniasis pathway (ko05140). **Figure S6D.** The systemic lupus erythematosus pathway (ko05322). Genes colored in red and blue represent that they were up-regulated in male and female magpies, respectively, and those colored in green indicate that expressions of the genes were either up- or down-regulated in both male and female magpies. (ZIP 121 kb)

## Data Availability

RNA-seq raw reads were available at the NCBI Sequence Read Archive under accession number SRP171777.

## Abbreviations

COG: Clusters of Orthologous Groups
DEG: differentially expressed gene
FPKM: fragment per kilobase of exon per million reads mapped
GO: gene ontogeny
KEGG: Kyoto Encyclopedia of Genes and Genomes
NCBI nr: National Center for Biotechnology Information nonredundant database
SSR: simple sequence repeats
Swiss-Prot/UniProtKB: Universal Protein resource, a central repository of protein data created by combining the Swiss-Prot, TrEMBL and PIR-PSD databases
