# Supplementary figures and images for: Transcriptome analysis revealed potential mechanisms of differences in physiological stress responses between caged male and female magpies
Source: BMC Genomics. 2019 Jun 3;20:447. doi: 10.1186/s12864-019-5804-0 (PMC6547487; doi:10.1186/s12864-019-5804-0)

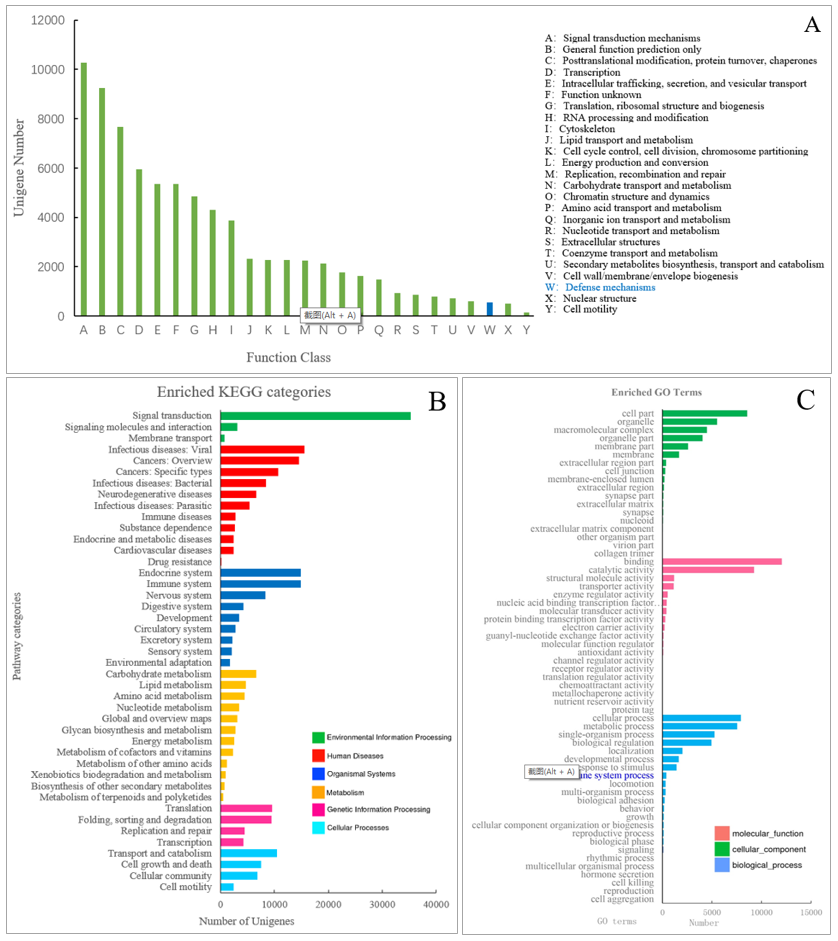

Supplement: Supplementary file 1 — Figure S1. Unigenes function categories. (A) COG function categories; (B) All unigenes KEGG barplot; (C) All unigenes Go Bar. (PNG 229 kb) [file 12864_2019_5804_MOESM1_ESM.png]

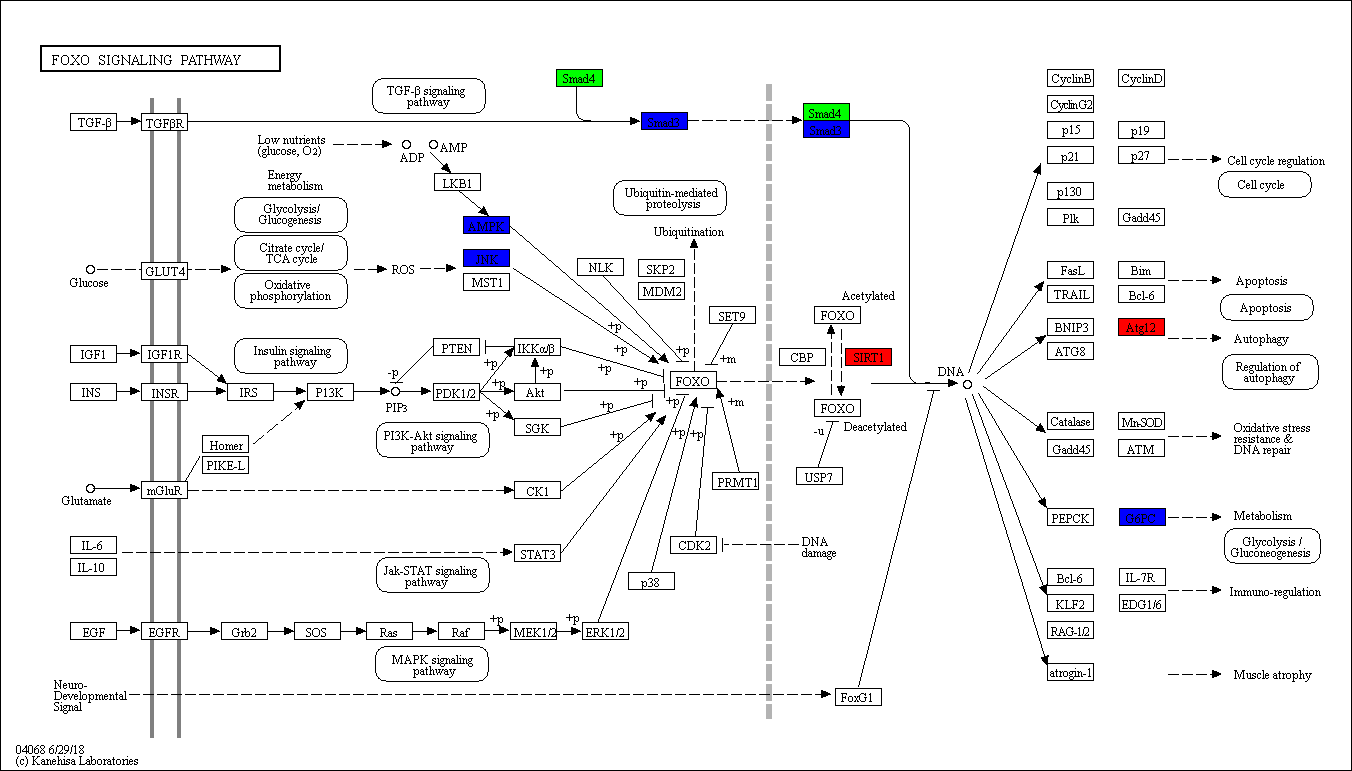

Supplement: Supplementary file 2 — FOXO signaling pathway (ko04068). Genes colored in red and blue represent that they were up-regulated in male and female magpies, respectively, and those colored in green indicate that expressions of the genes were either up- or down-regulated in both male and female magpies. (PNG 23 kb) [file 12864_2019_5804_MOESM2_ESM.png]

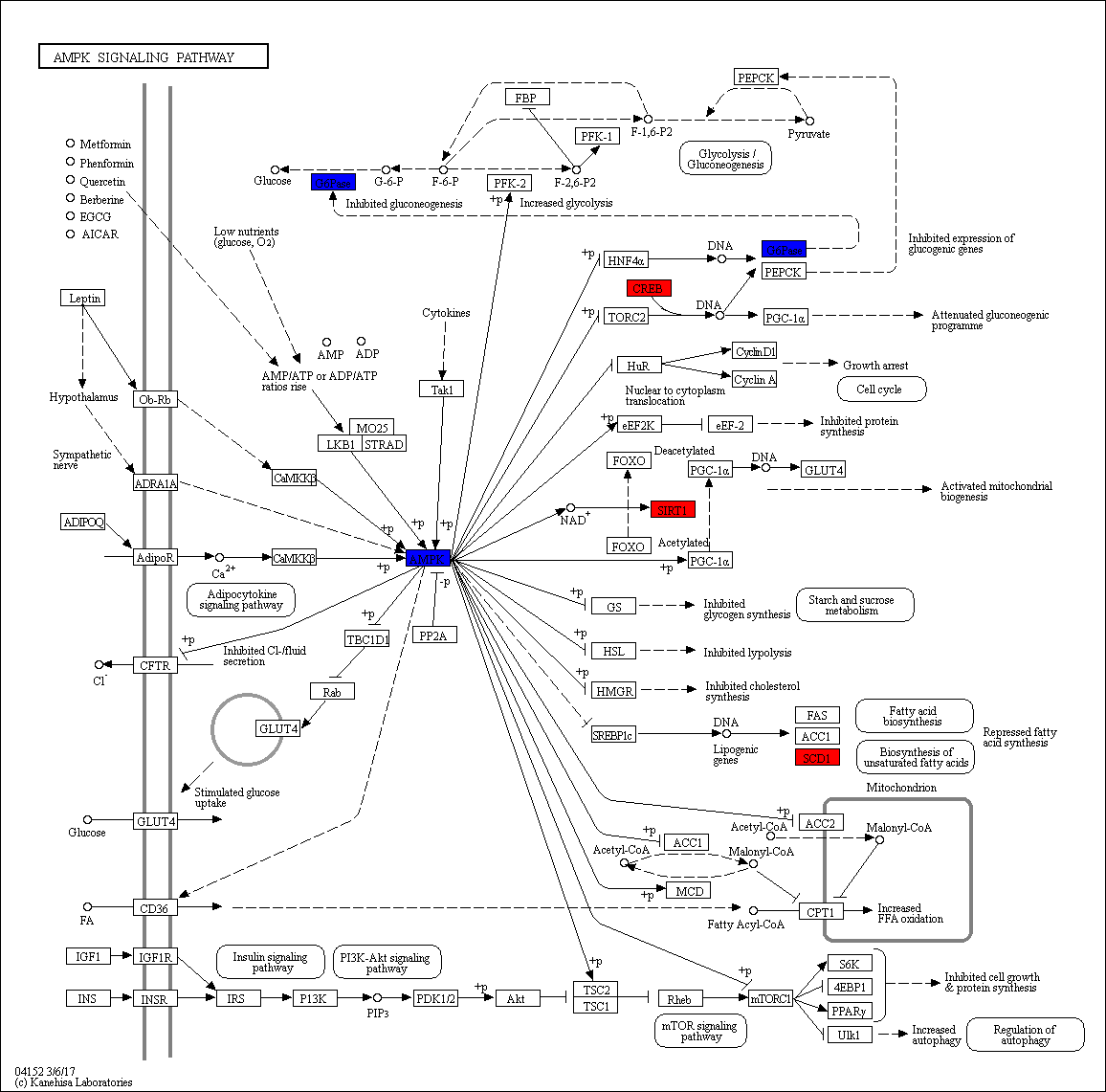

Supplement: Supplementary file 3 — The AMPK signaling pathway (ko04152). Genes colored in red and blue represent that they were up-regulated in male and female magpies, respectively, and those colored in green indicate that expressions of the genes were either up- or down-regulated in both male and female magpies. (PNG 33 kb) [file 12864_2019_5804_MOESM3_ESM.png]

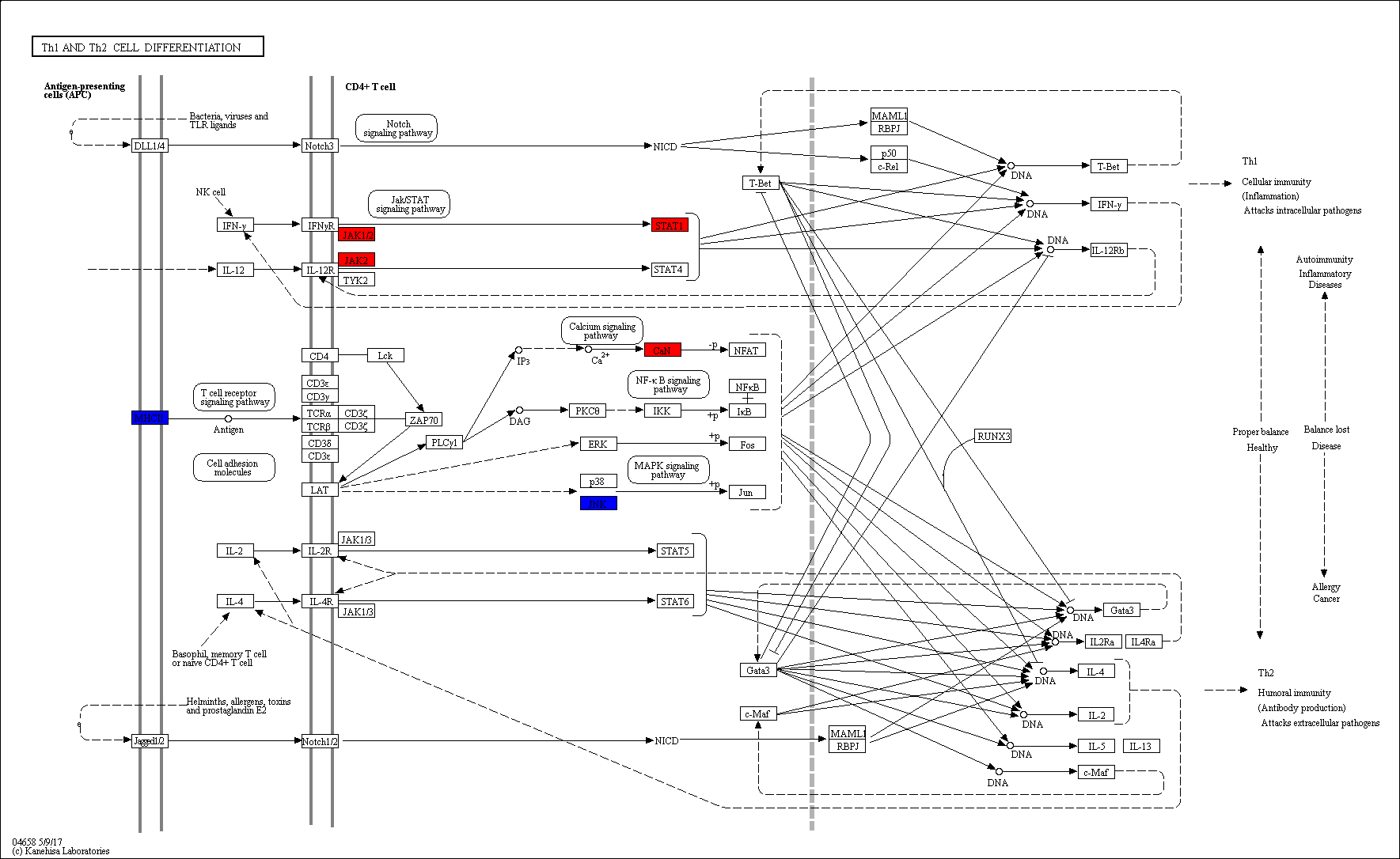

Supplement: Supplementary file 4 — Figure S4A. The Th1 and Th2 cell differentiation pathway (ko04658 ). Figure S4B. The Th17 cell differentiation pathway (ko04659 ). Genes colored in red and blue represent that they were up-regulated in male and female magpies, respectively, and those colored in green indicate that expressions of the genes were either up- or down-regulated in both male and female magpies. (ZIP 62 kb) [file 12864_2019_5804_MOESM4_ESM.zip › Fig. S4A Th1 and Th2 cell differentiation pathway (ko04658 ).png]

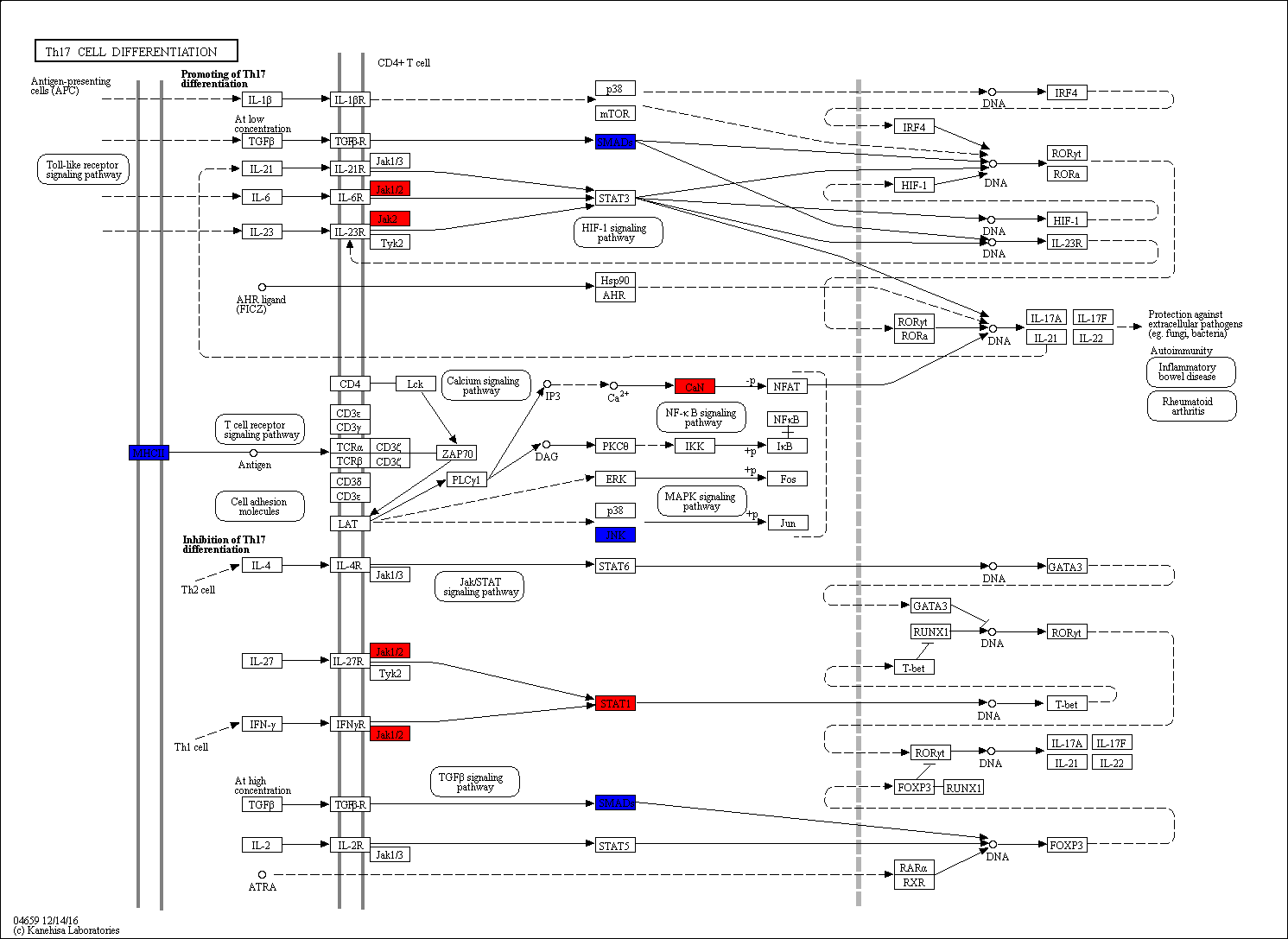

Supplement: Supplementary file 4 — Figure S4A. The Th1 and Th2 cell differentiation pathway (ko04658 ). Figure S4B. The Th17 cell differentiation pathway (ko04659 ). Genes colored in red and blue represent that they were up-regulated in male and female magpies, respectively, and those colored in green indicate that expressions of the genes were either up- or down-regulated in both male and female magpies. (ZIP 62 kb) [file 12864_2019_5804_MOESM4_ESM.zip › Fig. S4B Th17 cell differentiation pathway (ko04659 ).png]

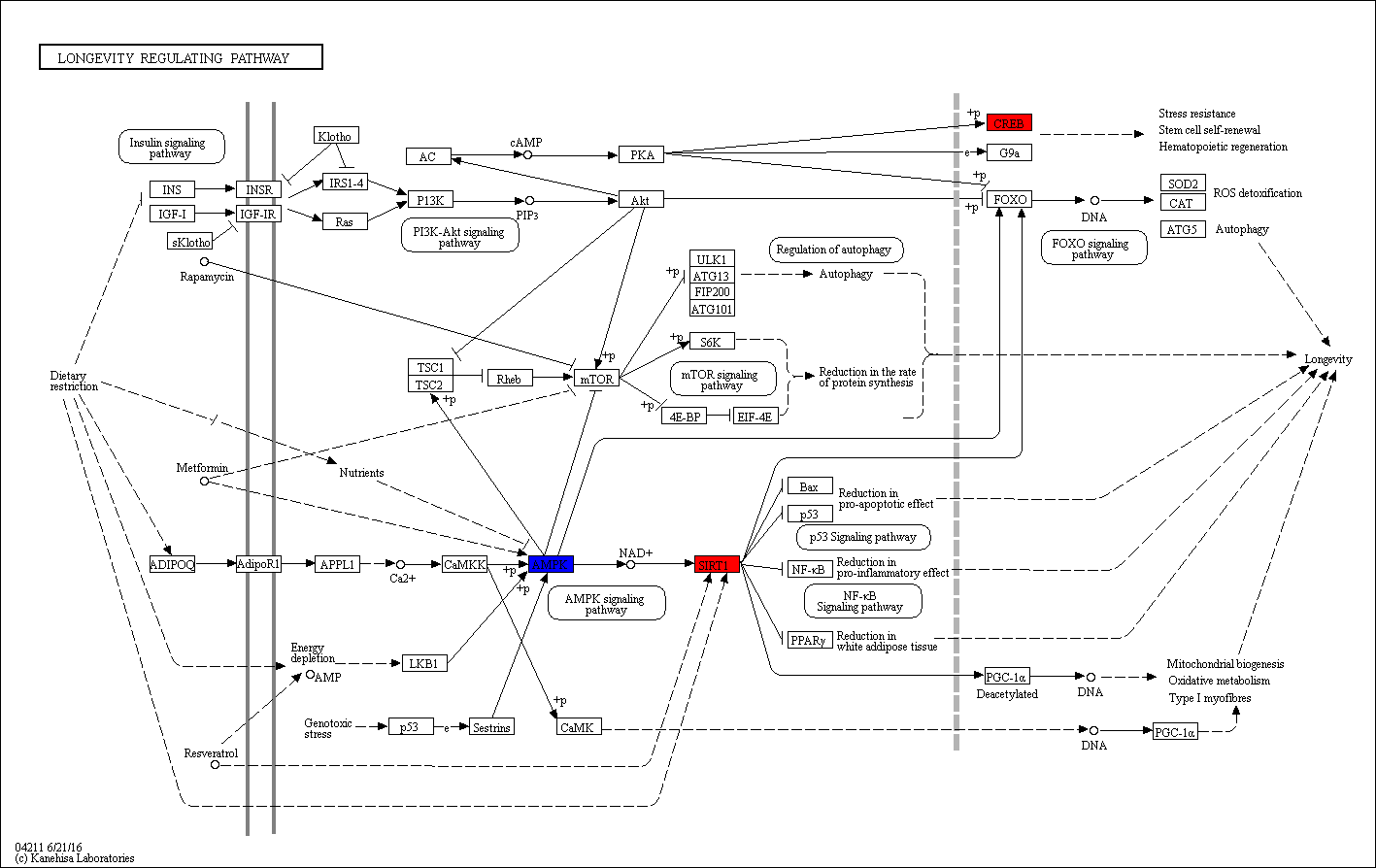

Supplement: Supplementary file 5 — Figure S5A. The longevity regulating pathway (ko04211). Figure S5B. The longevity regulating pathway-worm (ko04212 ). Figure S5C. The insulin signaling pathway (ko04910). Figure S5D. The mTOR signaling pathway (ko04150). Genes colored in red and blue represent that they were up-regulated in male and female magpies, respectively, and those colored in green indicate that expressions of the genes were either up- or down-regulated in both male and female magpies. (ZIP 93 kb) [file 12864_2019_5804_MOESM5_ESM.zip › Fig. S5A Longevity regulating pathway (ko04211).png]

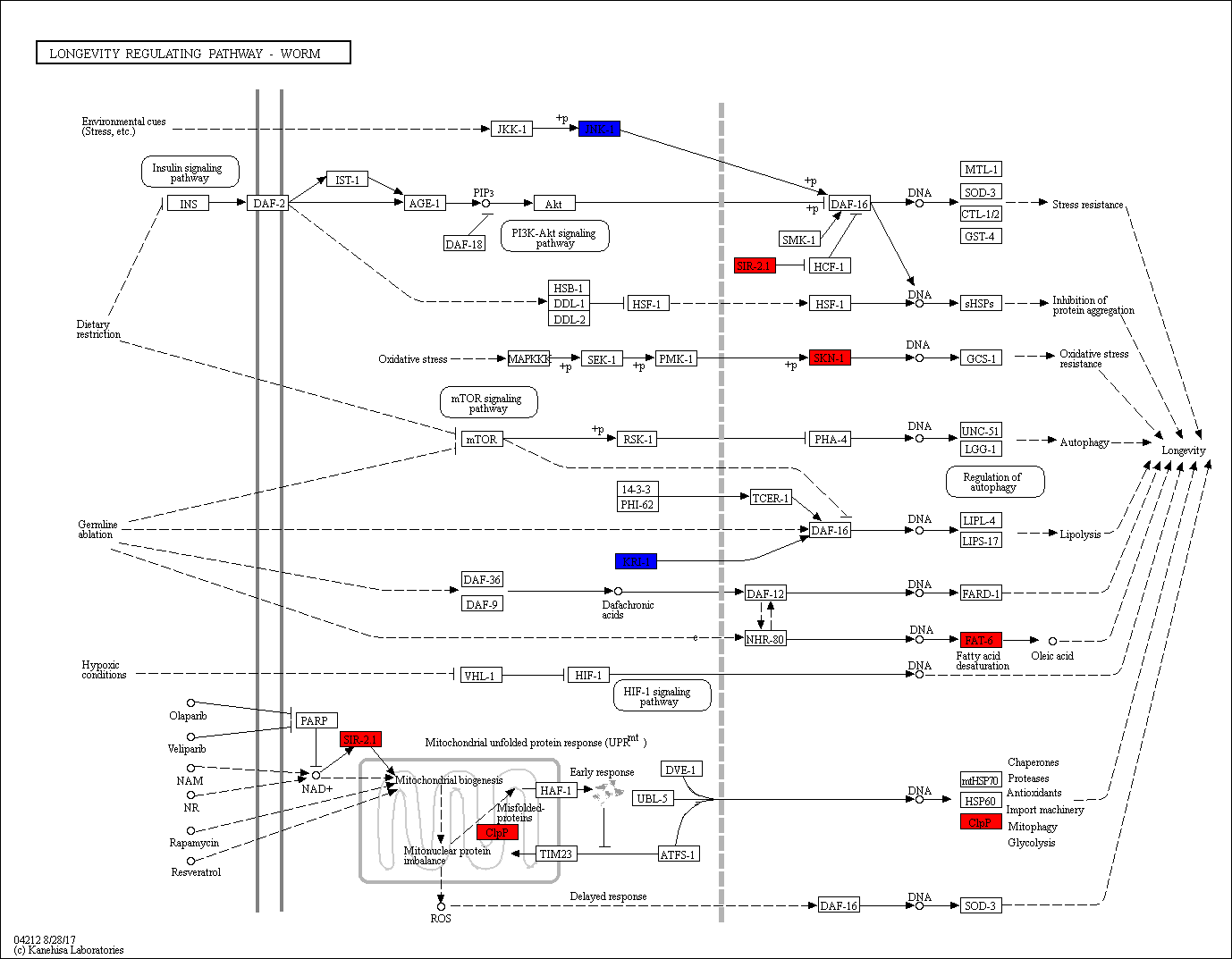

Supplement: Supplementary file 5 — Figure S5A. The longevity regulating pathway (ko04211). Figure S5B. The longevity regulating pathway-worm (ko04212 ). Figure S5C. The insulin signaling pathway (ko04910). Figure S5D. The mTOR signaling pathway (ko04150). Genes colored in red and blue represent that they were up-regulated in male and female magpies, respectively, and those colored in green indicate that expressions of the genes were either up- or down-regulated in both male and female magpies. (ZIP 93 kb) [file 12864_2019_5804_MOESM5_ESM.zip › Fig. S5B Longevity regulating pathway-worm (ko04212 ).png]

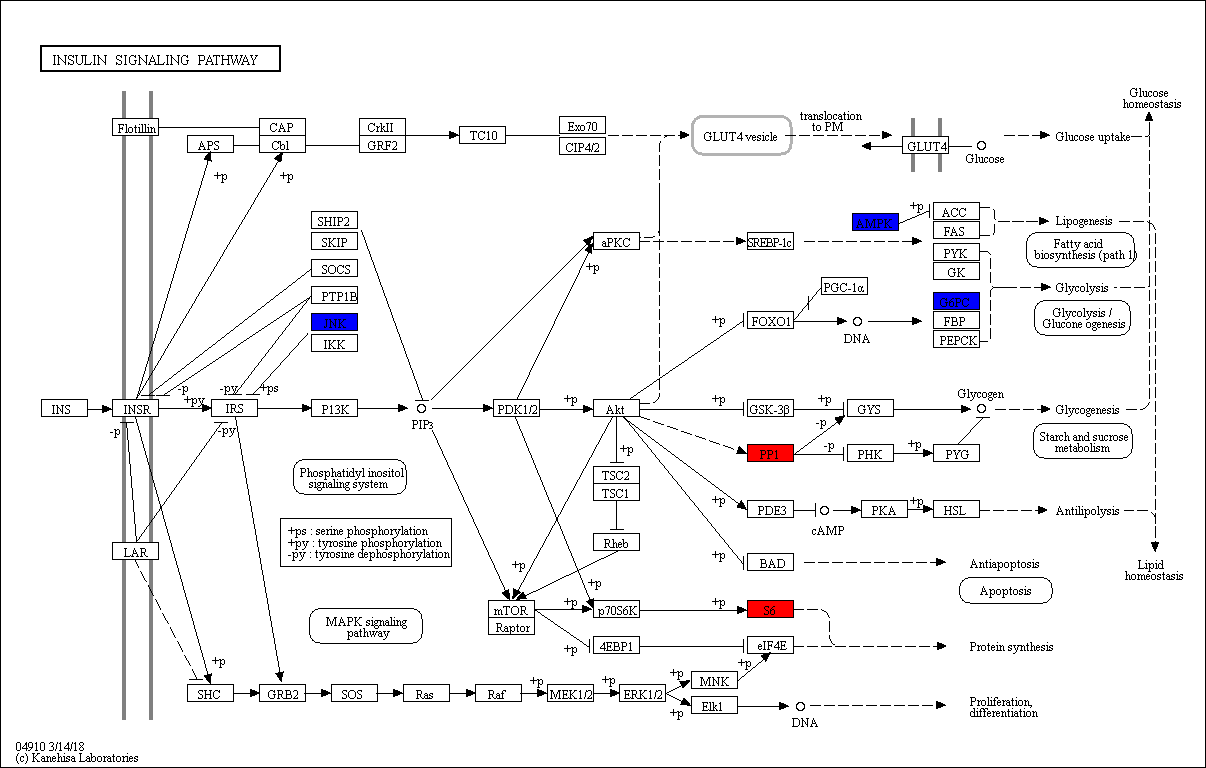

Supplement: Supplementary file 5 — Figure S5A. The longevity regulating pathway (ko04211). Figure S5B. The longevity regulating pathway-worm (ko04212 ). Figure S5C. The insulin signaling pathway (ko04910). Figure S5D. The mTOR signaling pathway (ko04150). Genes colored in red and blue represent that they were up-regulated in male and female magpies, respectively, and those colored in green indicate that expressions of the genes were either up- or down-regulated in both male and female magpies. (ZIP 93 kb) [file 12864_2019_5804_MOESM5_ESM.zip › Fig. S5C ko04910 Insulin signaling pathway.png]

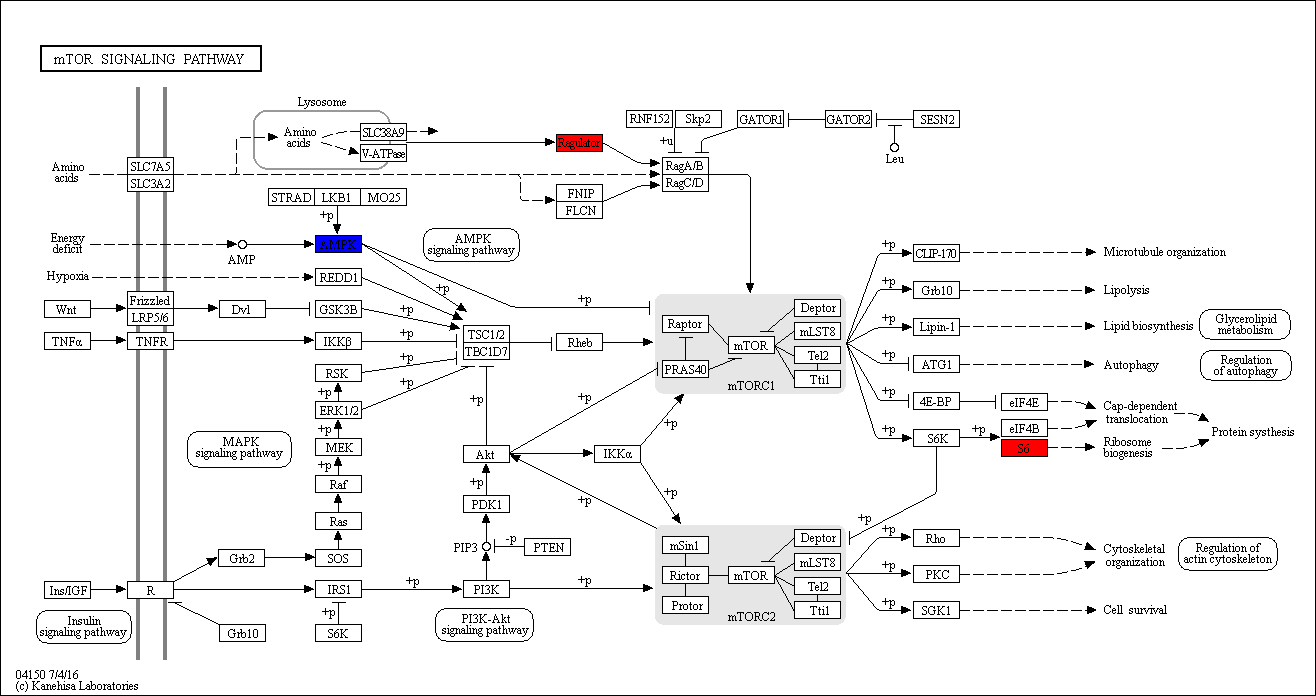

Supplement: Supplementary file 5 — Figure S5A. The longevity regulating pathway (ko04211). Figure S5B. The longevity regulating pathway-worm (ko04212 ). Figure S5C. The insulin signaling pathway (ko04910). Figure S5D. The mTOR signaling pathway (ko04150). Genes colored in red and blue represent that they were up-regulated in male and female magpies, respectively, and those colored in green indicate that expressions of the genes were either up- or down-regulated in both male and female magpies. (ZIP 93 kb) [file 12864_2019_5804_MOESM5_ESM.zip › Fig. S5D ko04150 mTOR signaling pathway.png]

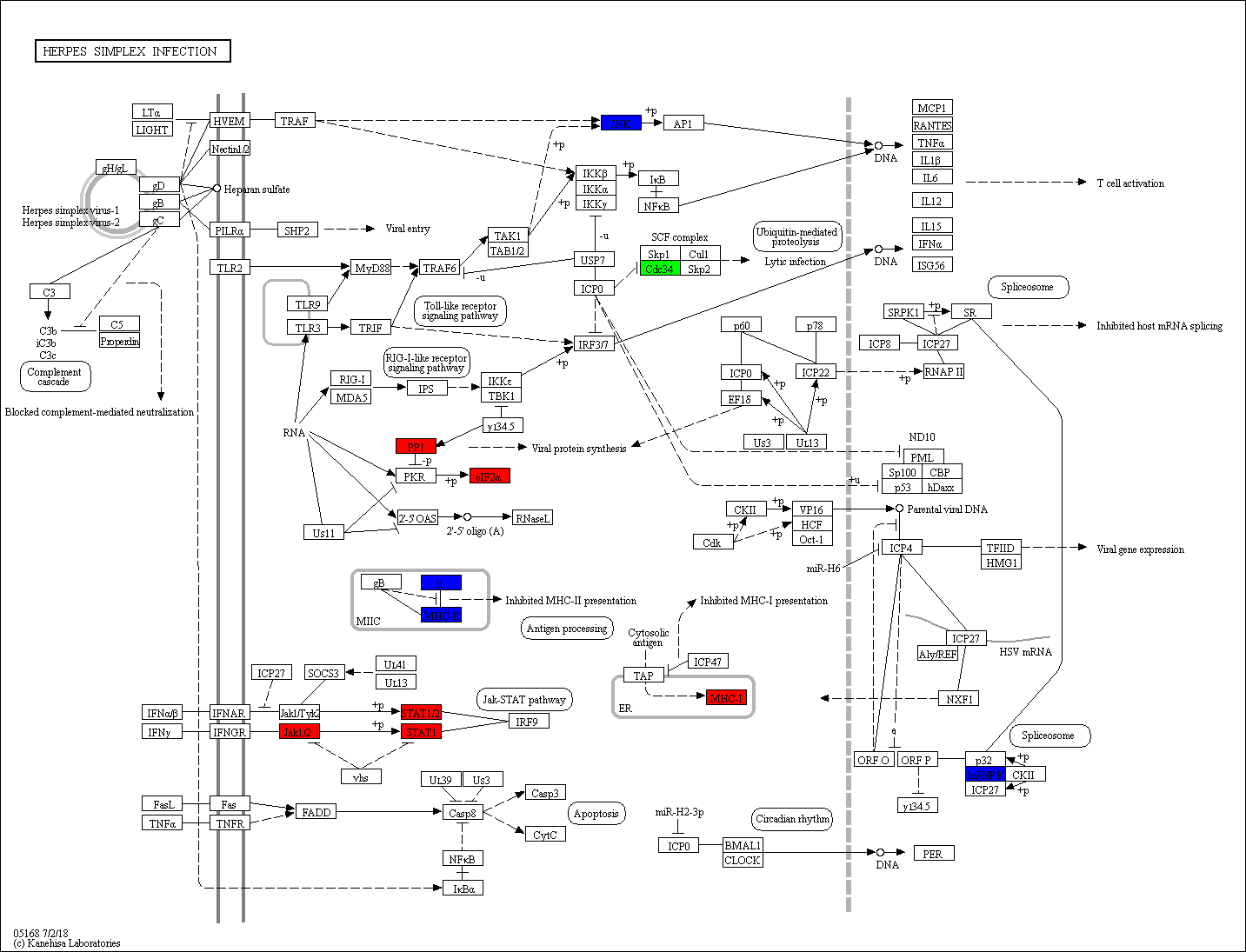

Supplement: Supplementary file 6 — Figure S6A. The herpes simplex infection pathway (ko05168). Figure S6B. The tuberculosis pathway (ko05152). Figure S6C. The leishmaniasis pathway (ko05140). Figure S6D. The systemic lupus erythematosus pathway (ko05322). Genes colored in red and blue represent that they were up-regulated in male and female magpies, respectively, and those colored in green indicate that expressions of the genes were either up- or down-regulated in both male and female magpies. (ZIP 121 kb) [file 12864_2019_5804_MOESM6_ESM.zip › Fig. S6A Herpes simplex infection pathway (ko05168).png]

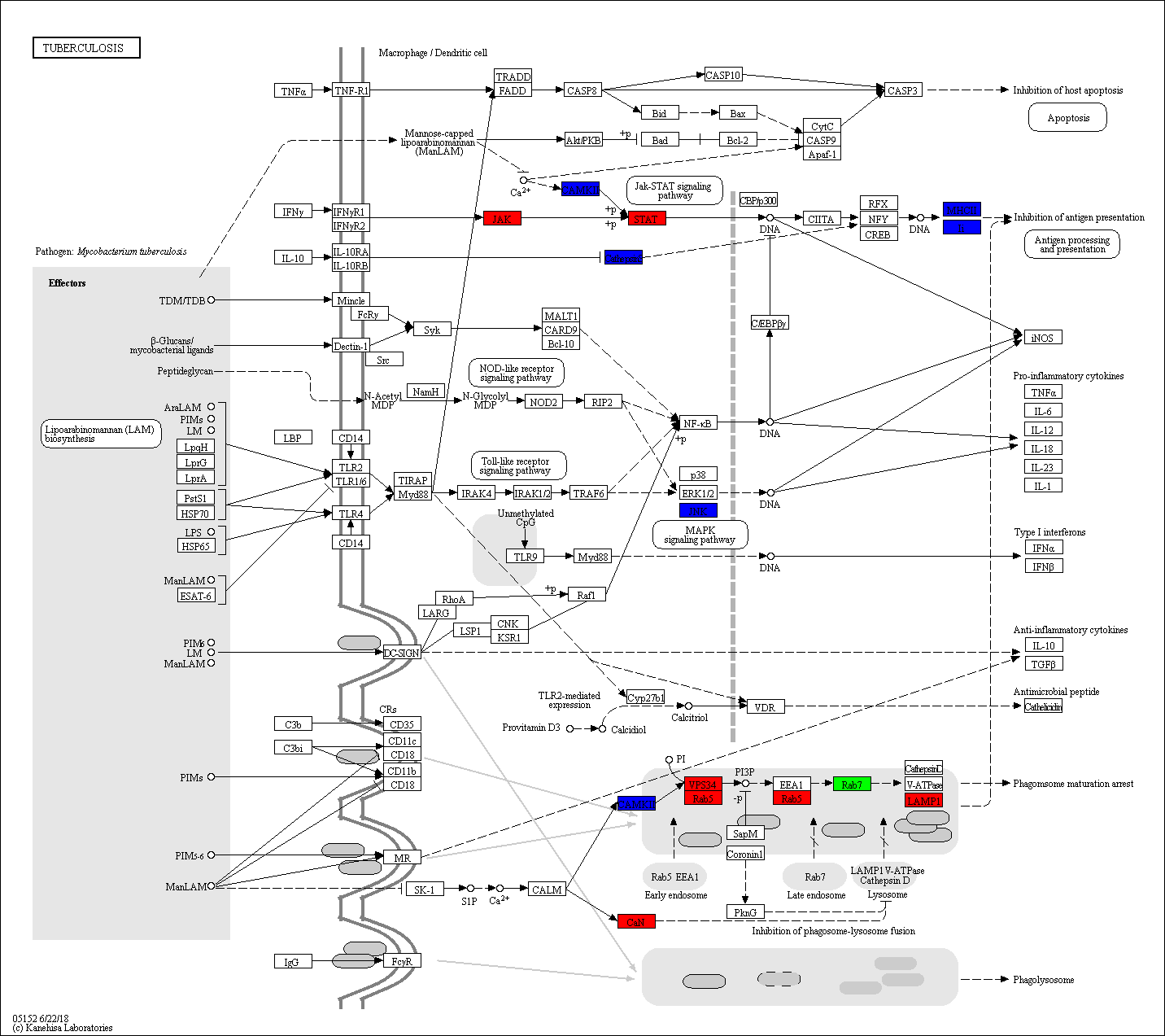

Supplement: Supplementary file 6 — Figure S6A. The herpes simplex infection pathway (ko05168). Figure S6B. The tuberculosis pathway (ko05152). Figure S6C. The leishmaniasis pathway (ko05140). Figure S6D. The systemic lupus erythematosus pathway (ko05322). Genes colored in red and blue represent that they were up-regulated in male and female magpies, respectively, and those colored in green indicate that expressions of the genes were either up- or down-regulated in both male and female magpies. (ZIP 121 kb) [file 12864_2019_5804_MOESM6_ESM.zip › Fig. S6B Tuberculosis (ko05152).png]

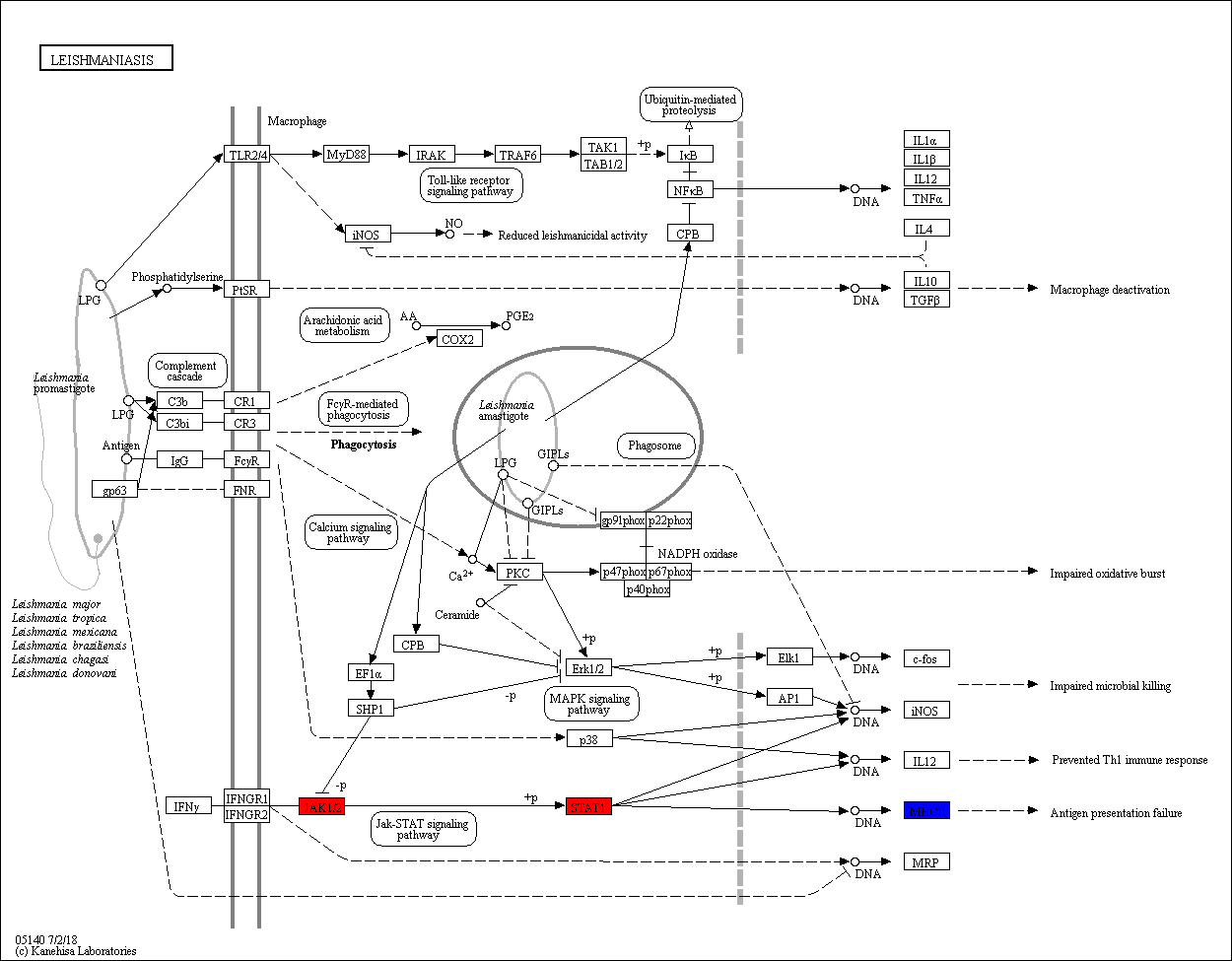

Supplement: Supplementary file 6 — Figure S6A. The herpes simplex infection pathway (ko05168). Figure S6B. The tuberculosis pathway (ko05152). Figure S6C. The leishmaniasis pathway (ko05140). Figure S6D. The systemic lupus erythematosus pathway (ko05322). Genes colored in red and blue represent that they were up-regulated in male and female magpies, respectively, and those colored in green indicate that expressions of the genes were either up- or down-regulated in both male and female magpies. (ZIP 121 kb) [file 12864_2019_5804_MOESM6_ESM.zip › Fig. S6C Leishmaniasis pathway (ko05140).png]

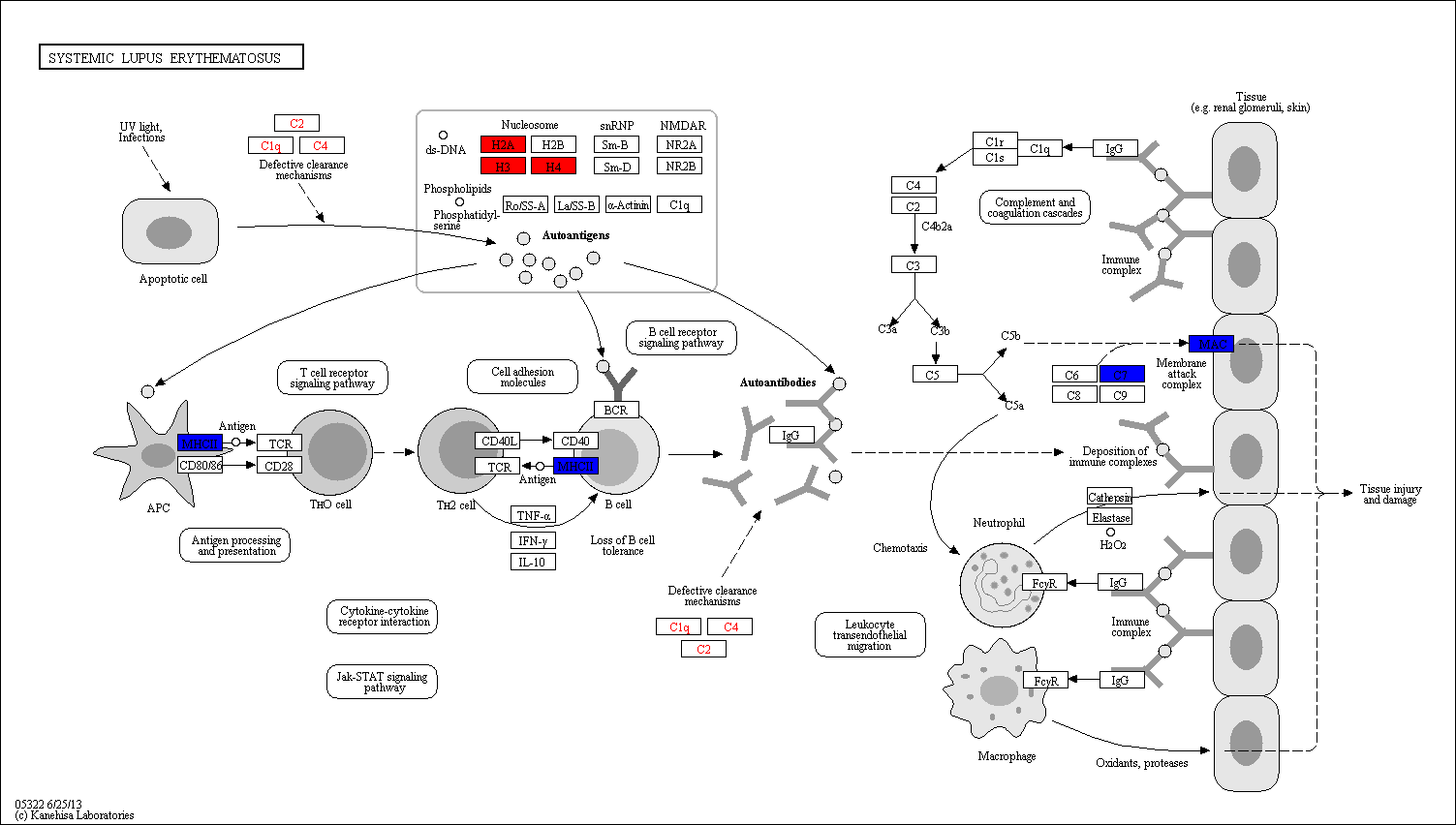

Supplement: Supplementary file 6 — Figure S6A. The herpes simplex infection pathway (ko05168). Figure S6B. The tuberculosis pathway (ko05152). Figure S6C. The leishmaniasis pathway (ko05140). Figure S6D. The systemic lupus erythematosus pathway (ko05322). Genes colored in red and blue represent that they were up-regulated in male and female magpies, respectively, and those colored in green indicate that expressions of the genes were either up- or down-regulated in both male and female magpies. (ZIP 121 kb) [file 12864_2019_5804_MOESM6_ESM.zip › Fig. S6D Systemic lupus erythematosus pathway (ko05322).png]
